# Morphometrical, Morphological, and Immunocytochemical Characterization of a Tool for Cytotoxicity Research: 3D Cultures of Breast Cell Lines Grown in Ultra-Low Attachment Plates

**DOI:** 10.3390/toxics10080415

**Published:** 2022-07-24

**Authors:** Fernanda Malhão, Ana Catarina Macedo, Alice Abreu Ramos, Eduardo Rocha

**Affiliations:** 1 Laboratory of Histology and Embryology, Department of Microscopy, ICBAS–School of Medicine and Biomedical Sciences, University of Porto (U.Porto), Rua de Jorge Viterbo Ferreira n° 228, 4050-313 Porto, Portugal; fcmalhao@icbas.up.pt (F.M.); acfpmacedo@gmail.com (A.C.M.); ramosalic@gmail.com (A.A.R.); 2 Histomorphology, Physiopathology, and Applied Toxicology Team, Interdisciplinary Center for Marine and Environmental Research (CIIMAR/CIMAR), University of Porto (U.Porto), Avenida General Norton de Matos s/n, 4450-208 Matosinhos, Portugal

**Keywords:** 3D cell culture, breast cancer, model characterization, light and electron microscopy

## Abstract

Three-dimensional cell cultures may better mimic avascular tumors. Yet, they still lack characterization and standardization. Therefore, this study aimed to (a) generate multicellular aggregates (MCAs) of four breast cell lines: MCF7, MDA-MB-231, and SKBR3 (tumoral) and MCF12A (non-tumoral) using ultra-low attachment (ULA) plates, (b) detail the methodology used for their formation and analysis, providing technical tips, and (c) characterize the MCAs using morphometry, qualitative cytology (at light and electron microscopy), and quantitative immunocytochemistry (ICC) analysis. Each cell line generated uniform MCAs with structural differences among cell lines: MCF7 and MDA-MB-231 MCAs showed an ellipsoid/discoid shape and compact structure, while MCF12A and SKBR3 MCAs were loose, more flattened, and presented bigger areas. MCF7 MCAs revealed glandular breast differentiation features. ICC showed a random distribution of the proliferating and apoptotic cells throughout the MCAs, not fitting in the traditional spheroid model. ICC for cytokeratin, vimentin, and E-cadherin showed different results according to the cell lines. Estrogen (ER) and progesterone (PR) receptors were positive only in MCF7 and human epidermal growth factor receptor 2 (HER-2) in SKBR3. The presented characterization of the MCAs in non-exposed conditions provided a good baseline to evaluate the cytotoxic effects of potential anticancer compounds.

## 1. Introduction

Human cancer cell lines are largely used to study the disease mechanisms and in vitro drug screening. Most of these studies are based on two-dimensional (2D) cell cultures in which cells grow in a monolayer adhered to a flat plastic surface [1,2]. This structural geometry is associated with fewer cellular interactions leading to a restricted cellular microenvironment that is translated into more restricted biochemistry, gene expression, and drug metabolism [3,4,5]. The strategy provides a model with limited predictive capacity, especially in drug discovery [3,6,7]. Therefore, there has been a growing interest in upgrading the 2D culture models to approach them to the in vivo solid tumors. Three-dimensional (3D) culture techniques have emerged as an attempt to mimic the physiological conditions of tumors and therefore bridge the gap between 2D and in vivo studies [2,3].

Traditionally, 3D cell cultures are described as an agglomerate of cells with a spherical shape, characterized by a decreasing gradient of nutrients, growth factors, oxygen, and pH values from the surface to the core [8,9]. This cell line culture configuration encompasses three zones: (1) an outer proliferative zone (where cells have direct access to the oxygen and nutrients); (2) an intermediate zone with quiescent cells; (3) an inner necrotic center due to the lack of nutrients and oxygen [2,10,11,12].

The arrangement of cells into 3D aggregates favors their functionality [4,13,14,15]. While morphological differentiation can be restored, cell multilayers promote diffusion gradients with heterogeneous populations of dividing, quiescent and dead cells, thus mimicking well nonvascular tumors [3,11,16]. These multilayers also constitute a barrier to the penetration of compounds [11], offering better predictions of the in vivo drug efficacy and toxicity [17]. Additionally, more cell-to-cell interactions promote higher intercellular networks and stimulate the production of extracellular matrix (ECM) proteins [6]. All the mentioned differences between 2D and 3D cultures explain why the former provides insufficient or inappropriate information and may sometimes overestimate the efficacy of some potential antineoplastic drugs [8,15,18].

There are several techniques for generating 3D cell cultures, but they overall fall into two main approaches: (a) scaffold-based, where cells are cultivated in synthetic or biological matrices; (b) scaffold-free, where cells are cultivated only in a culture medium, using, e.g., hanging drop or low attachment plates [19,20]. Different methodologies can generate different 3D cell aggregates that vary in size, shape, density, compactness, surface features, and internal structure [21,22].

One of the simplest methods for obtaining 3D cultures is the forced floating method [23]. This technique uses non-adhesive surfaces, such as the ultra-low attachment (ULA) plates with U-shaped bottomed wells or other surfaces coated with hydrophilic substances (e.g., agar or poly-Hema), to minimize cell–substrate adhesion, and forcing cells to remain suspended. A centrifugation step is commonly included to help cells aggregate and form spheroids (one per well) [24,25]. These plates are described to generate reproducible spheroids with uniform size and shape, suitable for high-throughput drug screening [1].

Breast cancer (BC) is the most commonly diagnosed cancer among women in western countries [26,27] and a leading cause of cancer death [28]. Male BC is rare (1% of all cancer in men), but the prognosis is much worse than in women [29]. BC comprises different biological subtypes with distinct histological and molecular characteristics, implying different therapeutic approaches and clinical outcomes [30,31]. The main differences in the molecular subtypes rely on the presence or absence of estrogen (ER) and progesterone receptors (PR), and the overexpression of the oncogene human epidermal growth factor receptor 2 (HER-2) [31]. Given the needs, new drugs continue to be investigated (particularly for the most lethal triple-negative BC) and translated from the laboratory to patients [32].

Considering the presented scenario, the use of 3D cell cultures of breast cell lines can be a very useful tool in the screening of anticancer compounds. Some authors have already reported the formation of 3D cultures from different cell lines, including BC ones, using ULA 96-well plates [22,33,34,35]. However, there are quite different results reported in the literature, especially about their morphology. These differences can be related to the characteristics of the cell line, and to the applied culture methodologies that many times are not described in detail hampering the investigators to reproduce them. Furthermore, we agree with other researchers who call for the need for a detailed morphofunctional and phenotypic characterization of the 3D models to better understand the results and properly compare studies [3,22,36,37].

For this study, three human BC cell lines were selected to represent the main BC subtypes: (1) MCF7: ER^+^, PR^+^, and HER-2^−^, corresponding to the most common BC type-Luminal A, (2) SKBR3: ER^−^, PR^–^, and HER-2^+^ representing the HER-2 BC subtype [38,39], and (3) MDA-MB-231, a “triple-negative cell line” (ER^−^, PR^−^, HER-2^−^), corresponding to the basal type breast carcinoma cell [38,40]. Additionally, we included a non-tumoral breast cell line (4) MCF12A [38,40], as some studies also include non-tumoral cells when screening new drugs or studying the toxicity of known compounds [41].

Given the above, the objectives of this study were: (1) to describe in detail the protocols and give some technical tips for the formation and analysis of MCAs from the four mentioned human breast cell lines using the ULA plates, and (2) to characterize the MCAs using morphometry, descriptive morphology (at light and electron microscopy) and quantitative immunocytochemistry (% labeled cells). This baseline characterization under no exposure conditions may offer a good starting point to evaluate the possible cytotoxic effects of compounds including potential anticancer ones.

## 2. Materials and Methods

### 2.1. Cell Lines and Culture Conditions

The MCF7 cell line was purchased from the European Collection of Authenticated Cell Cultures (ECACC, London, UK). MCF12A, MDA-MB-231, and SKBR3 cell lines were acquired from American Tissue Culture Collection (ATCC, Massanas, VA, USA). Cell lines were cultivated in T75 cm^2^ culture flasks in an MCO 19AIC (Sanyo, Osaka, Japan) incubator with 5% CO_2_ at 37 °C and subcultured at 80–90% confluence. MCF7, MDA-MB-231, and SKBR3 cells were cultivated with Dulbecco’s modified Eagle’s medium-high glucose (DMEM) without glutamine and phenol red, supplemented with 10% fetal bovine serum (FBS) (*v*/*v*) and 1% penicillin/streptomycin (100 U/mL/100 μg/mL, respectively). MCF12A cells were cultivated in Dulbecco’s modified Eagle’s medium/Ham’s nutrient mixture F12 (DMEM/F12) medium supplemented with 20 ng/mL human epidermal growth factor (EGFR), 100 ng/mL cholera toxin, 0.01 mg/mL insulin and 500 ng/mL hydrocortisone, 10% FBS and 1% penicillin/streptomycin.

### 2.2. Cell Culture Reagents and Wares

Cholera toxin, insulin, human epidermal growth factor, and hydrocortisone (Sigma Aldrich, St. Louis, MO, USA). DMEM without glutamine and phenol red, Trypsin/EDTA, penicillin/streptomycin, and FBS (Biochrom KG, Berlin, Germany). DMEM/F12 medium without phenol red (GE Healthcare, Chicago, IL, USA). All other used reagents and chemicals were analytical grade. T75 cm^2^ flasks were from (Orange Scientific, Braine-l’Alleud, Belgium) and ULA plates (Corning, New York, MA, USA).

### 2.3. 3D Cell Culture Procedure

Cell suspensions were obtained by trypsinization of confluent T75 cm^2^ flasks using 0.25% trypsin/0.02% EDTA, at 37 °C until cell detachment. Trypsin was inactivated by adding twice the volume of the complete fresh medium. Cell suspensions were counted using a Neubauer chamber and then cells were seeded in 96-well ULA plates (200 µL per well). In a preliminary test, cells were seeded at different cell densities: MCF7 and SKBR3-10, 20, and 40 × 10^4^ cells/mL; MDA-MB-231-20, 40 and 50 × 10^4^ cells/mL and MCF12A 5, 10 and 20 × 10^4^ cells/mL. The plates were centrifuged with a Rotina 380 R (Hettich, Tuttlingen, Germany), at 200× *g* for 10 min, at room temperature, and then placed in an incubation chamber with 5% CO_2_ at 37 °C. A 7-day experiment was made, using 3 days (72 h) for MCA formation [24,42], then the medium was replaced by a culture medium with 0.1% DMSO [24], simulating a potential drug/compound exposure for more 4 days (96 h). On day 3, we collected MCAs of 2 independent replicas to compare their morphology with MCAs collected on day 7.

### 2.4. Study Design

Nine independent replicas were performed, each one corresponding to one 96-well microplate. The same MCAs were measured and paired at the two time points (days 3 and 7 in culture). The number of MCAs per replica was dependent on the studied parameter: (a) 12 MCAs of each cell line per replica were used for morphometry (108 MCAs measured per cell line); (b) 3 MCAs of each cell line per replica were harvested for cytological and immunocytochemical analysis at light microscopy; (c) 2 MCAs of each cell line per replica were harvested for transmission electron microscopy (TEM).

### 2.5. Morphological Analysis

#### 2.5.1. MCA Area Measurements

Each MCA (one per well) was photographed with an SZX10 stereomicroscope (Olympus, Tokyo, Japan) linked to a DP21 digital camera (Olympus, Tokyo, Japan), at two time points: day 3 (before removing medium for exposure simulation) and day 7 (after the simulation of 96 h of exposure)—immediately before sampling. The images were submitted to the AnaSP freeware [43] for area measurements.

Data were statistically analyzed using PAST 3.1 [44] and GraphPad Prism 6 (GraphPad Software, San Diego, CA, USA). Data obtained from MCAs areas and corresponding to nine independent replicates were presented as mean ± standard deviation (SD). Data sets were tested for normality (Shapiro–Wilk test) and homogeneity of variances (Levene test) and then paired Student t-tests were applied to compare the area variation of the same MCAs between days 3 and 7. The significance level was set at 5%.

#### 2.5.2. Cytological Analysis at Light Microscopy

##### Processing for Paraffin Embedding

First, the culture medium was removed from the wells containing the MCAs, and then 200 µL/well of 10% buffered formalin (Bioptica, Milan, Italy) was gently added and left for 20 min. This brief first fixation step into the wells helped to prevent the disintegration of the MCAs during their collection. Then, the MCAs were transferred to Eppendorf microtubes containing the same fixative, using a P1000 micropipette with a sectioned tip to augment the diameter of the tip to prevent damaging the MCAs. After 24 h fixation, MCAs were embedded in histogel (Thermo Scientific, Waltham, MA, USA), according to the manufacturer’s instructions, to avoid the loss of MCAs during processing for paraffin embedding. Next, histogel containing MCAs was placed into tissue cassettes and processed using an automatic tissue processor Leica TP120 (Leica, Nussloch, Germany). The processing protocol consisted of 1 h in each of the following sequence of reagents: ethanol (70%, 90%, 95%, and 100% (twice); xylene: ethanol (1:1); xylene (twice); liquid paraffin (twice)). Paraffin blocks were performed in an embedding station EG 1140H (Leica, Nussloch Germany). Three-micrometer thickness sections were obtained in a Leica 2255 microtome (Leica, Nussloch Germany) and placed onto silane-treated KP-frost slides (Klinipath, Duiven, Nederland). For paraffin melting, slides were placed for 20 min at 60 °C and then kept overnight at 37 °C. They were used either for standard hematoxylin-eosin (HE) staining or immunocytochemistry (ICC).

##### HE Staining

MCA sections were deparaffinized in xylene (2 × 10 min) and hydrated following a descendent sequence of ethanol (100%, 95%, 70%) and running tap water, 5 min each. Nuclei were stained with Mayer’s hematoxylin (Merck, Darmstadt, Germany) for 3 min, and then slides were washed in running tap water for 5 min to remove the excess dye. Next, slides were immersed in aqueous 1% eosin Y for 5 min (Merck, Darmstadt, Germany), followed by quick dips into distilled water. Lastly, slides were dehydrated in an ascending series of ethanol (95%, 100%, 100%), 5 min each, cleared in xylene (2 × 5 min), and coverslipped using Coverquik 2000 medium (VWR Chemicals, Briari, France).

#### 2.5.3. Immunocytochemistry (ICC)

For ICC, MCA sections were deparaffinized and hydrated as described for HE staining. Heat antigen retrieval was performed in a pressure cooker, where slides were immersed in boiling citrate buffer 0.01 M, pH 6.0. The cooker was then closed, and slides were left 3 min after reaching maximum pressure. After slowly cooling, endogenous peroxidase blocking was made with 3% hydrogen peroxide in methanol (10 min). The excess hydrogen peroxide was removed by washing twice (5 min each) in Tris-buffer saline pH 7.6 (TBS). Slides were dried around the sections (without leaving them to dry or damage), and the sections were circled with a hydrophobic pen (Leica Biosystems, Milton Keynes, UK). In sequence, unspecific reactions were blocked using the protein block-specific reagent for that of the Novolink™ Polymer detection kit (Leica Biosystems, Milton Keynes, UK) (5 min), followed by two washes in TBST (TBS with 0.05% of Tween 20). Primary antibodies were diluted in phosphate buffer saline (PBS) with 5% of bovine serum albumin and incubated overnight at 4 °C (corresponding to 16 h of incubation), using a humidified chamber (details of antibodies and used dilutions in Table 1).

For negative control, the primary antibody was substituted by antibody diluent only. According to antibody datasheet recommendations, the positive controls used corresponded to human tissues where the target antigens are expressed. 

The mentioned Novolink™ Polymer detection system was used for signal amplification, according to the manufacturer’s instructions, using the chromogen 3,3′-Diaminobenzidine (DAB). After the primary antibody incubation and after each step of the detection system, slides were washed twice (5 min each) in TBST. For nuclear counterstain, cells were immersed in Mayer’s hematoxylin (1 min), washed, then slides were dehydrated with an ascendant sequence of ethanol (90%, 95%, absolute ethanol (twice)), cleared in xylene, and mounted. Lastly, slides were observed with a light microscope BX50 (Olympus, Tokyo, Japan) and photographed with a DP21 digital camera (Olympus, Tokyo, Japan).

We selected a panel of antibodies targeting different outputs for the MCAs’ ICC characterization. First, we investigated the proliferating and death status of the cells within the MCAs using Ki67 and caspase-3 antibodies as markers of proliferation [45,46] and apoptosis [47,48], respectively.

Additionally, we evaluated the expression of the cell surface E-cadherin, a protein related to cell adhesion [49] and cell polarity [50]. Additionally, the epithelial-mesenchymal transition was evaluated by using the epithelial marker AE1/AE3 [51] and the mesenchymal marker vimentin [52].

Finally, we investigated the expression of the ER and PR and the overexpression of HER-2, both described as discriminative characteristics of the used breast cell lines [38,39,53].

#### 2.5.4. Immunocytochemistry Quantitative Analysis

For each tested antibody, three representative images corresponding to the central part (equator) of three random MCAs were analyzed. Estimation of the percentage of immunomarked cells was made by superimposing to the section a sampling grid with forbidden lines to prevent edge effects [54]. Only cells with a sectioned nucleus were counted. A minimum of 150 cells was counted per MCA, and a total of 500 cells were counted per cell line. Data were statistically analyzed using PAST 3.1 [44] and GraphPad Prism 6 (GraphPad Software, San Diego, CA, USA). Normality and homogeneity of variances were tested using the Shapiro–Wilk and Levene tests, respectively. Significant differences (*p* < 0.05) were assessed by one-way ANOVA, followed by the post hoc Holm–Šídák multiple comparison test.

#### 2.5.5. Transmission Electron Microscopy (TEM)

MCAs were collected as described for the analysis by light microscopy. After harvesting to Eppendorf microtubes, MCAs were fixed for 2 h in 2.5% glutaraldehyde in 0.15 M sodium cacodylate-HCl buffer, pH 7.2, 4 °C, and then washed (2 × 10 min) in the same buffer. Post fixation was carried out using a 1% osmium tetroxide solution in the mentioned buffer for 2 h at 4 °C, followed by two washes of 10 min. MCAs were then sequentially dehydrated (30 min in each reagent) in: 50%; 70%; 95%, 100% ethanol (twice); propylene oxide (30 min, twice). For epoxy resin embedding, we used consecutive mixtures consisting of different parts of propylene oxide and epoxy resin, respectively, (3:1); (1:1); (1:3), and ultimately only resin (1 h each), to allow an optimal and gradual resin penetration in the MCAs. After embedding in rubber molds, they were placed in the oven at 60 °C for 48 h for resin polymerization. Semithin (1.25 µm thick) and ultrathin (90 nm thick) were obtained in an ultramicrotome EM UC7 (Leica, Nussloch, Germany). Semithin sections were collected onto silane-coated slides (Klinipath, Duiven, The Netherlands) and stained with a mixture of aqueous azure II and methylene blue (1:1), observed in a BX50 light microscope (Olympus, Tokyo, Japan) and photographed with a DP21 digital camera (Olympus, Tokyo, Japan). Ultrathin sections were obtained with a diamond knife (Diatome, Nidau, Switzerland), placed onto 200 mesh copper grids (Agar Scientific, Stansted, UK), and contrasted with 3% aqueous uranyl acetate (20 min) and Reynold’s lead citrate (10 min). TEM observations used a 100CXII microscope (Jeol, Tokyo, Japan), operated at 60 kV, equipped with an Orius SC1000 CCD digital camera (Gatan, Pleasanton, CA, USA), controlled by the Digital Micrograph software (Gatan, Pleasanton, CA, USA).

## 3. Results

### 3.1. MCA Area Measurements

**Figure 1:**
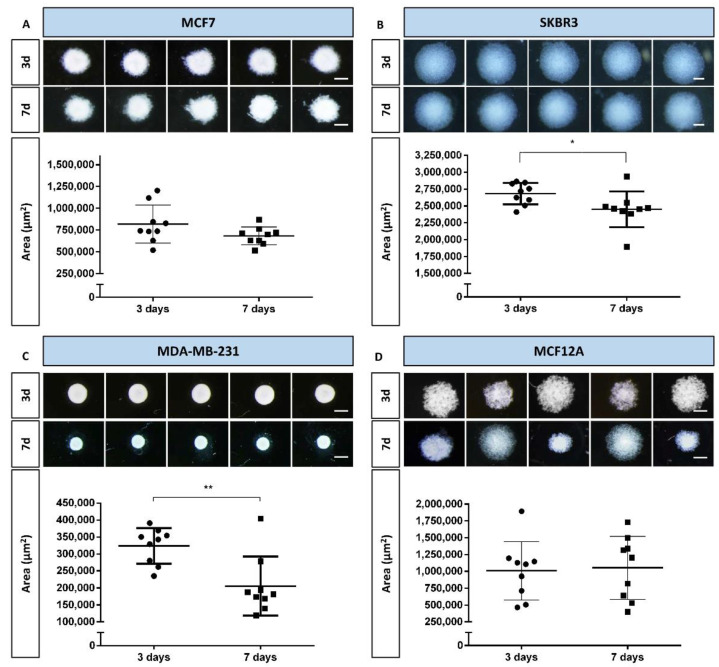
Representative stereomicroscopic images and areas (measured by AnaSP software) of multicellular aggregates (MCAs) of the four cell lines, on days 3 and 7: (**A**) MCF7, (**B**) SKBR3, (**C**) MDA-MB-231, and (**D**) MCF12A. Areas are expressed as mean ± standard deviation of nine independent experiments. Each dot in the graphs corresponds to the mean area of one replicate. Significant differences: * *p* < 0.05, ** *p* < 0.01. Scale bar: 500 µm.

After the preliminary test with different cell densities, the densities that corresponded to better formation of MCAs were selected for each cell line (see representative images of the MCAs in Figure S1). The selected cell densities were: 40 × 10^4^ cells/mL (MCF7, MDA-MB-231, and SKBR3) and 20 × 10^4^ cells/mL (MCF12A). The MCAs were observed daily, and images were captured on days 3 and 7 of culture. Figure 1 summarizes the key aspects of MCAs from all cell lines, showing their morphology using comparative images of the same MCAs on the two sampling days. It includes graphs of their areas, highlighting the variance among replicates, making it easier to compare morphology with size changes. 

During the first days of culture, cells aggregated progressively, and on day 3 all the cell lines formed MCAs (see Figure 1). On day 3, stereomicroscopic observation showed that the MCAs were spherical (Figure 1), the MDA-MB-231 aggregates being the ones that most resembled a perfect sphere (Figure 1C). However, when the MCAs were manipulated to change the culture medium (at day 3), it became clear that the actual shape of the 3D structures was more of an ellipsoid (an oblate spheroid) than of a sphere, especially in the SKBR3 and MCF12A cell lines.

Irrespective of the number of days in culture, SKBR3 MCAs were the largest ones, followed, in decreasing order, by the MCF12A, MCF7, and MDA-MB-231 MCAs. The size of the MCAs was related to the degree of compactness, as the larger MCAs were also the looser ones, where cells were not so closely attached (SKBR3 and MCF12A, Figure 1B and D), while the smaller MCAs were more compact, containing cells that were more tightly packed (MCF7 and MDA-MB-231, Figure 1A,C). Additionally, MDA-MB-231 displayed well-defined borders, contrary to other cell lines where the limits were more irregular; as it is noticeable comparing Figure 1C with Figure 1A,B,D.

When comparing MCAs from days 3 and 7, it was possible to observe some changes in size and morphological appearance. In MDA-MB-231 MCAs, there was clear compaction, resulting in a statistically significant area reduction (Figure 1C). Although not as visually evident, a statistically significant decrease in areas of MCAs was also noted in SKBR3 MCAs (Figure 1B).

The most variable MCAs were the ones from the MCF12A cell line, on both 3 and 7 days, because the areas would either increase or decrease, as noted by the large amplitude of the standard deviations and morpho-phenotype (Figure 1D).

### 3.2. Histological Analysis

**Figure 2:**
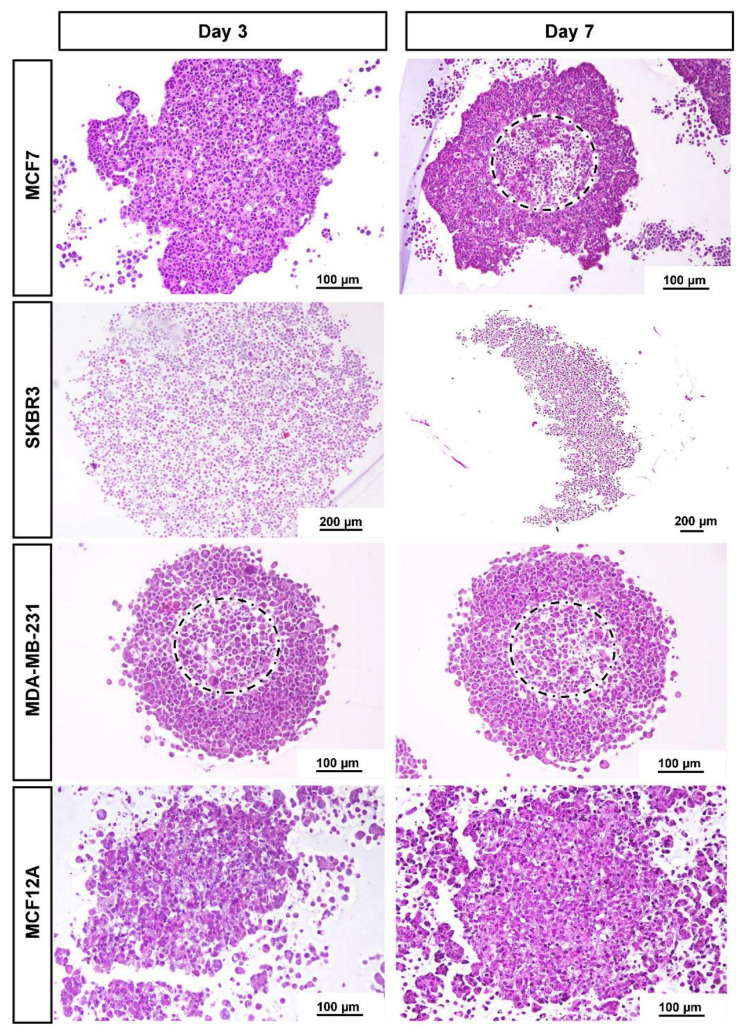
Representative histological images of MCAs of the four cell lines: MCF7, SKBR3, MDA-MB-231, and MCF12A stained with HE on days 3 and 7 in culture. MCF7 and MDA-MB-231 formed compact MCAs, while SKBR3 and MCF12A formed loose MCAs. The dashed circles limit the apoptotic/necrotic core in compact MCAs.

Figure 2 presents the typical morphology of the MCAs cultured for 3 and 7 days, at the light microscopic level. In line with the stereomicroscopicanalysis, cell compactness in the MCAs varied among cell lines. MCF7 and MDA-MB-231 were more compact, while SKBR3 and MCF12A were not tightly packed. There was a notable space among SKBR3 cells, contrasting with the more cell-tightened MCAs. Besides the loose structure, SKBR3 MCAs were quite resistant to manipulation. The same was not true for MCF12A MCAs, since they tend to partially or totally disintegrate when manipulated. Regarding these MCAs, the inner core stayed more attached, while the outer part tended to disaggregate, showing dispersed cells around the central region.

When sections reached nearly the core of the compact MCAs harvested on both days 3 or 7, it was observed in some MCAs the presence of an apoptotic/necrotic zone of different sizes (dashed circles in Figure 2). The central core had “empty” spaces and a higher number of cells with hyperchromatic and pycnotic nuclei, hypereosinophilic cytoplasm, and apoptotic bodies. In parallel, some cells presented nuclear swelling and pale cytoplasm. In contrast, the looser MCAs did not present apoptotic/necrotic cores.

The general morphology of the MCAs from each cell line, observed with light microscopy, was similar on the two sampled days (days 3 and 7). As we wanted to evaluate the MCAs in the context of an experimental setting of exposure to a compound of interest (3 days for MCAs formation plus 4 days of exposure), further characterization data are only given after 7 days in culture. The MCF7 MCAs presented some unique features that are described along with the ICC and TEM results.

### 3.3. Immunocytochemical Characterization

**Figure 3:**
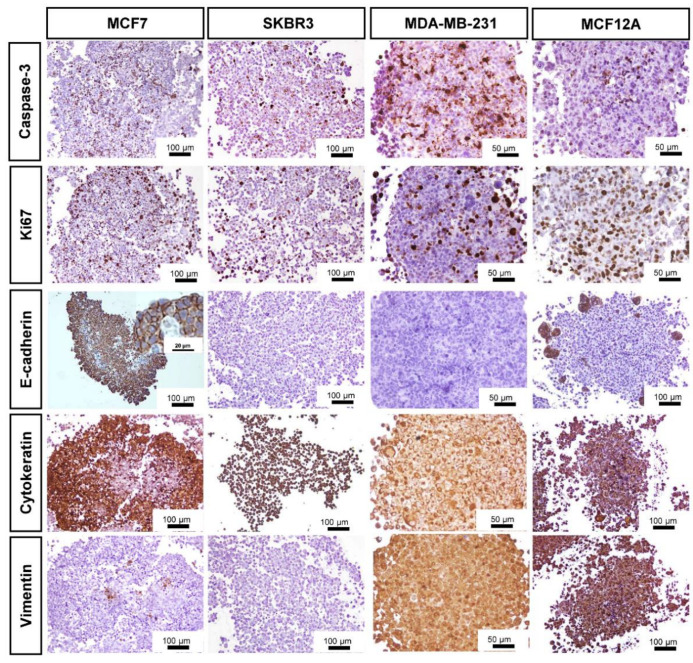
Representative images of ICC characterization of MCAs from MCF7, SKBR3, MDA-MB-231, and MCF12A after 7 days in culture. Brown staining with diaminobenzidine (DAB) indicates positive staining at different cellular parts according to the antigen localization: Caspase-3: nucleus and cytoplasm; Ki67: nucleus; E-cadherin: cell membrane; Cytokeratin AE1/AE3: cytoplasm; Vimentin: cytoplasm.

The distribution of the immunostaining was easily seen in low magnification images (Figure 3).

All MCAs displayed cells immunostained for caspase-3, varying on average from 15 to 30%. The positively stained caspase-3 cells were randomly distributed throughout the whole MCAs from all cell lines. However, when an apoptotic/necrotic core was present in MCF7 and MDA-MB-231 MCAs, a high number of caspase-3 positive cells existed in the central region; despite the immunostaining reaching the outer cell layers. In MCF7 MCAs there were additional caspase-3 positive cells inside the lumen of acinar-like structures.

The pattern of Ki67 immunostaining was similar to that of caspase-3. All MCAs showed scattered proliferating cells (at the periphery, middle part, and core). Even when an apoptotic/necrotic core was present, there were some proliferating cells inside it.

Other MCAs immunophenotypes were assessed, such as the expression of the tumor suppressor protein E-cadherin. MCF7 MCAs highly expressed E-cadherin (ca. 70%), except in the central region when an apoptotic/necrotic core was present. In this case, cells changed their morphology and acquired characteristics of death, losing the E-cadherin immunomarking. MCF12A MCAs expressed E-cadherin in small groups of peripheral cells (ca. 11%). The SKBR3 and MDA-MB-231 were negative for E-cadherin.

Regarding the cytokeratin AE1/AE3 (epithelial marker), most cells in MCF7 MCAs were positive (ca. 76%). However, in the presence of an apoptotic/necrotic core, the cells lost the positivity for that marker, similarly to what was described for E-cadherin tagging in MCF7. Differently, in SKBR3, MCF12A, and MDA-MB-231 MCAs, all cells were positive for AE1/AE3. 

As to the intermediate filament protein vimentin, all cells were positive in MDA-MB-231 and MCF12A MCAs. In contrast, in MCF7 MCAs just a few cells stained positive (ca. 5%), and in SKBR3 MCAS no cells were tagged (Figure 3).

**Figure 4:**
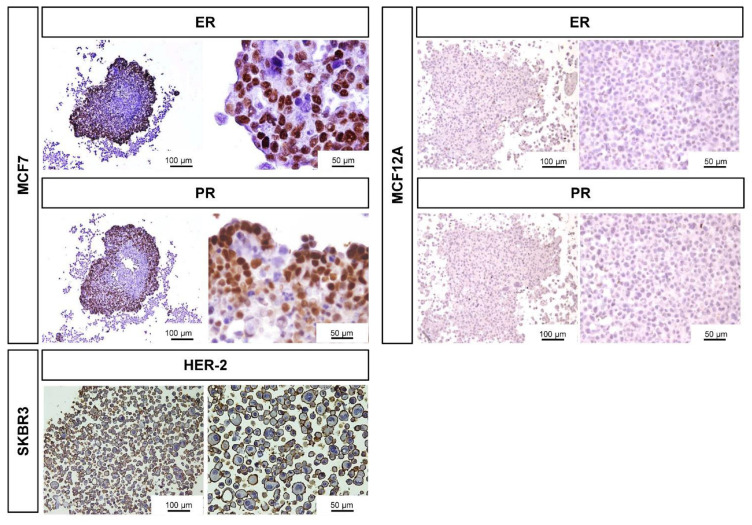
Representative images of ICC characterization of MCAs relative to the expression of ER, PR in MCF7 and MCF12A MCAs, and HER-2 staining in SKBR3 MCAs, after 7 days in culture. The brown DAB staining corresponds to the immunolocalization of the tested antibodies according to the antigen localization: ER/PR: nucleus; HER-2: membrane.

Additionally, we checked the ER/PR expression in the MCF7 and MCF12A MCAs, as well as the expression of the growth-promoting protein HER-2 in SKBR3 (Figure 4). Revealed by the nuclear brown staining, MCF7 MCAs had an average of 53% of their cells positively tagged for ER while 49% were stained for PR. The labeled cells were preferentially located in the outer part of the MCAs, where the intensity of the immunostaining was also stronger. In MCF12A MCAs, there were no positive cells for any of the hormone receptors. In SKBR3 MCAs, 82% of cells had the membrane totally stained for HER-2.

Table 2 summarizes the ICC quantification. For caspase-3 and Ki67 markers, we performed ANOVA statistical analysis to compare the different cell lines. There were no observed statistically significant differences in relation to caspase-3 positive cells. Concerning the percentage of proliferating cells stained with Ki67, the only statistical difference was observed between MCF7 and MCF12A (*p* < 0.05), MCF7 being more proliferative than MCF12A MCAs.

### 3.4. Specific Structural Features of MCF7 MCAs

**Figure 5:**
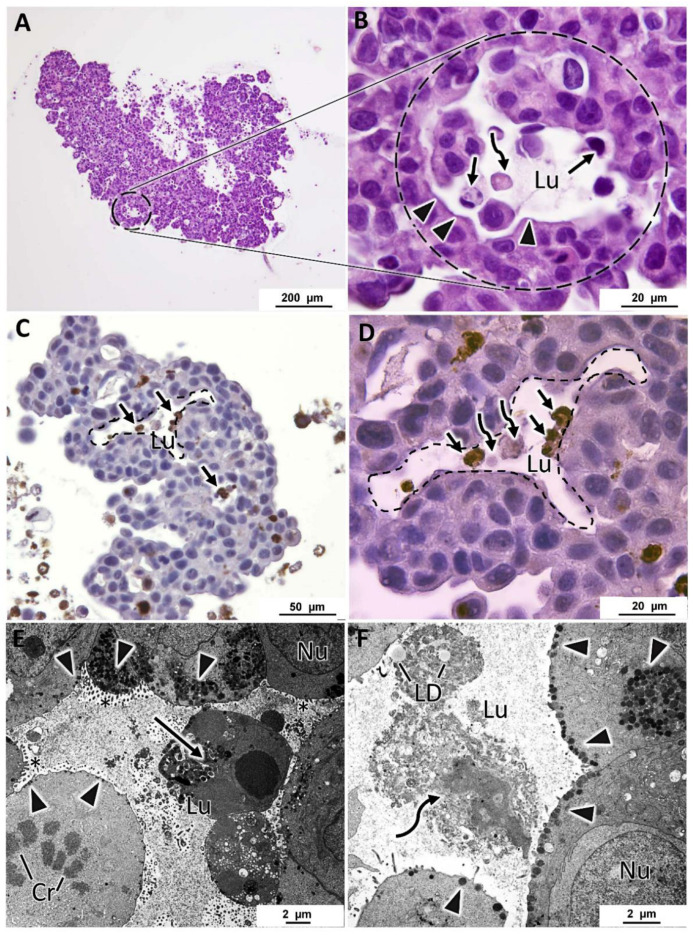
Representative aspects of acinar-like structures in MCF7 MCAs. (**A**,**B**) correspond to HE staining highlighting one acinar-like structure surrounded by a dashed circle. (**B**) is a higher magnification of (**A**) and shows apoptotic (arrows) and necrotic (curved arrows) cells within the lumen (Lu). Arrowheads evidence a basophilic line limiting the lumen. (**C**,**D**) ((**D**) is a higher magnification of (**C**)) correspond to ICC anti-caspase-3, acinar-like structures delimited with a dashed line; within the lumen, there are caspase-3 positive cells, stained in brown (arrows) and necrotic cells that do not stain with caspase-3 (curved arrows). (**E**,**F**) are TEM images showing acinar-like structures with a central lumen, containing apoptotic cells (arrow) in (**E**) and lipid droplets (LD) and necrotic cells (curved arrow) in (**F**). Cells show polarity, having microvilli towards the lumen (asterisks) and subplasmalema alignment of secretory vesicles (arrowheads). Cr: chromosomes in a mitotic cell; Nu: nucleus.

In all MCF7 MCAs, from days 3 and 7, it was noted many acinar-like structures were well visualized in HE staining (Figure 5A,B). In their lumina, they commonly had cells with structural features compatible with apoptosis, such as condensed hyperchromatic nuclei, nuclear fragmentation, and hypereosinophilic cytoplasm [55]. Additionally, there were cells with morphology compatible with necrotic cells presenting pale eosinophilic cytoplasm with a ghost cell aspect that at TEM corresponded to decreased electron density in the cytoplasm, loss of cell membrane integrity with leakage of cytoplasmatic content, organelle disruption, and nucleus dissolution morphology [55,56] (Figure 5B–D). The presence of apoptotic cells was confirmed by ICC against caspase-3 (Figure 5C,D). The cells of acinar-like structures revealed polarity, with microvilli in the apical pole (Figure 5E,F). The apical basophilic line (arrowheads in Figure 5B), that at TEM corresponded to long rows of secretory vesicles aligned towards the lumen (Figure 5E,F), reinforcing the presence of cell polarity.

### 3.5. General Ultrastructure of MCAs

**Figure 6:**
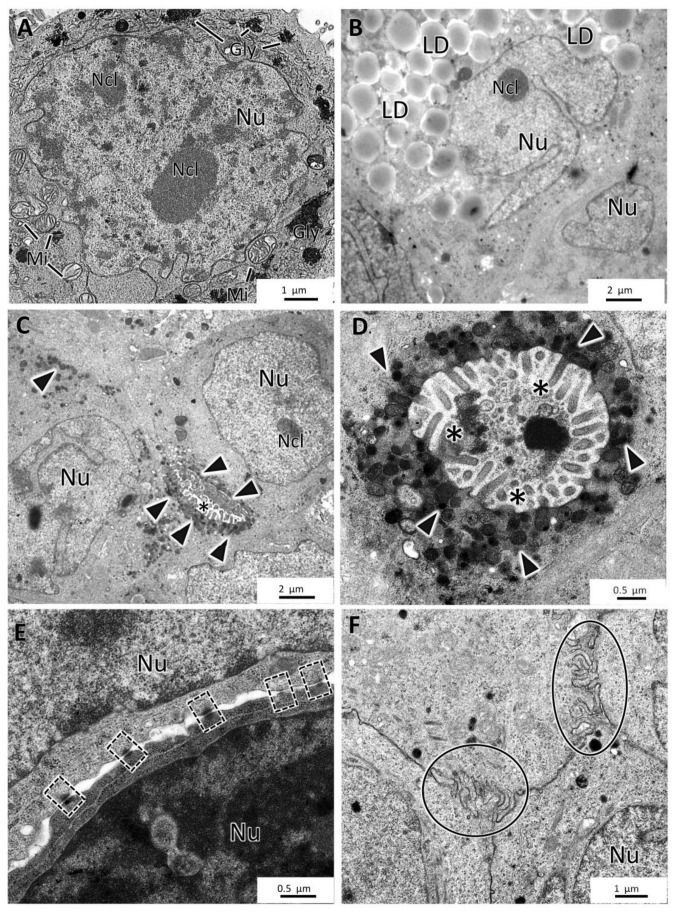
TEM representative images of MCF7 MCAs. (**A**,**B**) show storage of glycogen (Gly) and lipid droplets (LD), respectively. (**C**,**D**) display small intercellular canaliculi with microvilli (asterisks) and secretory vesicles (arrowheads). (**E**,**F**) highlight the presence of desmosomes (dashed-lined rectangles) and interdigitations (circles). Mi: mitochondria; Ncl: nucleoli; Nu: nucleus.

The four cell lines formed MCAs with different ultrastructural characteristics. MCF7 MCAs presented irregularly shaped nuclei with prominent nucleoli and round to elongated mitochondria. Rough endoplasmic reticulum and Golgi apparatus were rarely seen. The cytoplasm could show glycogen or lipids. Nevertheless, while some cells had neither glycogen nor lipids, others had moderate to high amounts of these substances (Figure 6A,B). There was no simultaneous presence of glycogen and lipid droplets within the same cell. 

Concomitantly with the recovery of cell polarity described above (Figure 5), small canaliculi, formed by the apposition of cells presenting features similar to acinar-like structures, were seen between some cells, also with microvilli and an accumulation of secretory vesicles towards the lumen (Figure 6C,D). MCF7 MCAs cells were closely attached, with many tight junctions, desmosomes, and interdigitations (Figure 6E,F).

**Figure 7:**
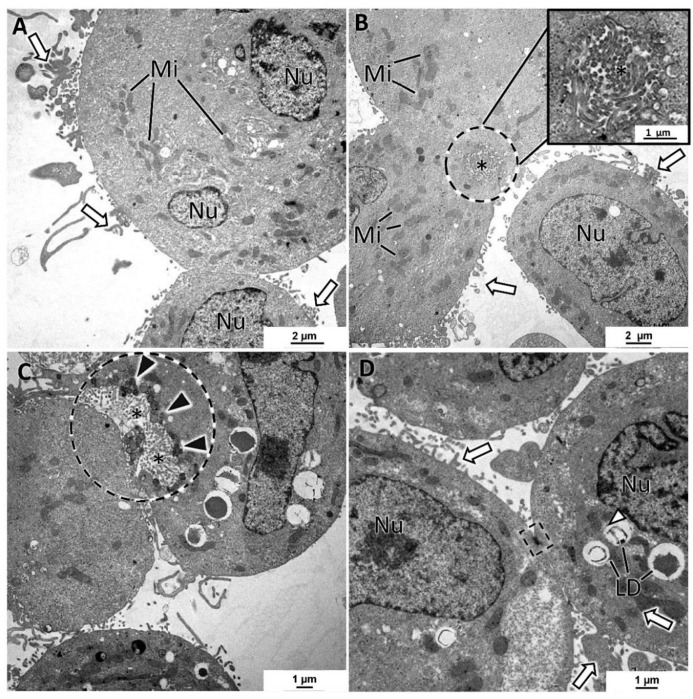
TEM representative images of SKBR3 MCAs. (**A**–**D**) show intercellular spaces with microvilli (white arrows). (**B**,**C**) evidence in the dashed-lined circles of the intercellular canaliculi formed by microvilli (asterisks) of adjacent cells. (**C**) depicts a canaliculus with some secretory vesicles nearby the lumen direction (black arrowheads). Cells presented different amounts of lipid droplets (LD) and were rich in mitochondria (Mi). Dashed-lined rectangles: desmosome; Ncl: nucleoli; Nu: nucleus.

SKBR3 MCAs were cell-loose structures, and this characteristic was evident in both HE and TEM sections. Cells with microvilli presented large intercellular spaces (white arrows) (Figure 7A–D). Nonetheless, some cells were attached, and among them, it was regularly observed small intercellular canaliculi bordered by the microvilli of adjacent cells (Figure 7B,C). The presence of secretory vesicles near these canaliculi was not so evident as in MCF7 MCAs, but in some canaliculi, they were present, although to a lesser extent (Figure 7C). Cells in the MCAs commonly had very irregularly shaped nuclei, prominent nucleoli, presented rare rough endoplasmic reticulum cisternae, and were rich in mitochondria. The most common storage substance was lipid droplets (Figure 7C,D). Desmosomes were rarely observed (Figure 7D).

**Figure 8:**
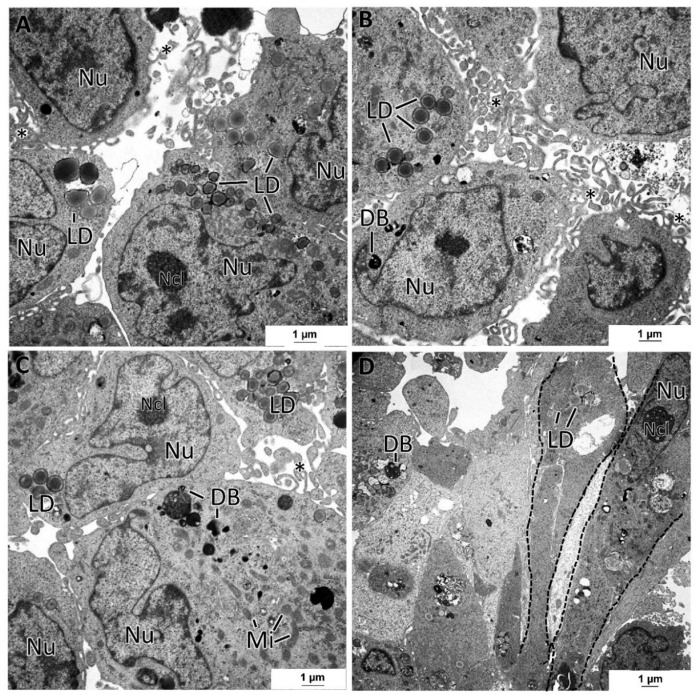
TEM representative images of MDA-MB-231 MCAs. (**A**–**D**) pictures illustrate cells displaying variable amounts of lipid droplets (LD) and dense bodies (DB). Intercellular spaces are circumscribed by cells displaying microvilli (asterisks). (**D**) shows some spindle cells (dashed lines). mitochondria: Mi; nucleus: Nu; nucleoli: Ncl.

The MDA-MB-231 MCAs revealed cells with irregular nuclei, many dense bodies, and lipid droplets (Figure 8A–D). The remaining cytoplasmic contents were scarce, with the Golgi cisternae and mitochondria being rarely seen. In some aleatory areas, cells showed tight cell-to-cell adhesions, while in other areas, cells were not so closely attached. At their surface, cells possessed a high number of membrane projections, forming areas of tangled microvilli projected to the intercellular spaces. A few cells presented an elongated spindle morphology (Figure 8D), contrasting with the usual roundish-to-ellipsoidal structure.

**Figure 9:**
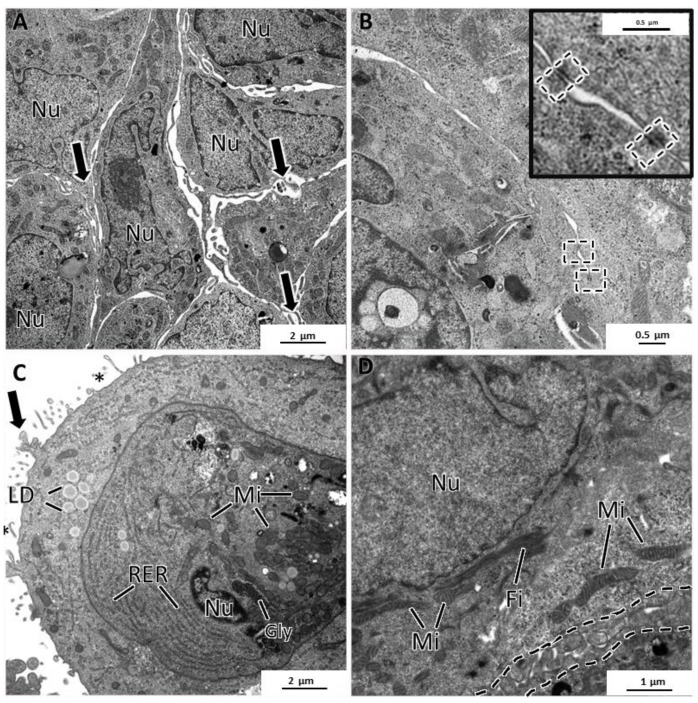
TEM representative images of MCF12A MCAs. (**A**) highlights intercellular spaces occupied by the cytoplasmatic projections on the cell’s surface (arrows). (**B**) shows some rare desmosomes (dashed rectangle). (**C**) depicts the cytoplasm content, rich in organelles such as mitochondria (Mi), rough endoplasmic reticulum (RER), storage substances, lipid droplets (LD), glycogen (Gly), microvilli (asterisks), and larger cytoplasmatic projections (arrow). (**D**) shows cytoplasmic filaments (Fi) arranged in bundles and the net of tangled short cytoplasmic projections (dashed line).

The MCF12A MCAs were characterized as having the lowest degree of intercellular adhesion. At TEM observation, cells almost did not adhere to each other and presented microvilli and larger cytoplasmatic projections on their surfaces directed to the intercellular spaces (Figure 9A). Despite the loose structure of the MCAs, desmosomes were present but were rarely observed (Figure 9B). Additionally, some cells were joined by a net of tangled membrane projections (Figure 9D). The MCF12A cells had the most irregular nuclei of all the studied MCAs and were the richest in organelle content. They had a profuse number of mitochondria and rough endoplasmic reticulum cisternae (Figure 9C). Additionally, it was common to see lipid droplets, small glycogen deposits (Figure 9C), and bundles of cytoplasmic microfilaments around the nucleus (Figure 9D) or dispersed in the cytoplasm. 

## 4. Discussion

This study detailed a single procedure to obtain MCAs of three BC cell lines from different molecular subtypes and one non-tumoral cell line using ULA plates. Furthermore, and to our best knowledge, we characterized for the first time those MCAs using morphometry, cytology (light and electron microscopy), and immunocytochemistry. We recorded some practical experiences from this study, which we have organized here as a set of “tips and tricks” to assist readers in effectively obtaining and analyzing MCAs using ULA plates (Table S1).

### 4.1. MCAs’ Formation in ULA Plates

This methodology enabled the generation of uniform MCAs (a single MCA per well) from all the cell lines within 3 days of culture, which accords with other authors that used the same methodology [11,22,57]. Another report described spheroids formation in 1 to 2 days [18], but although this time would be enough for the MDA-MB-231 cell line, it would not allow the spheroid formation for the other cell lines. Thus, to uniformize and perform the experiment in all cell lines at the same time, we used 3 days. Indeed, the adopted total of 7 days in culture (3 days of MCA formation plus simulation of 4 days of exposure) was similar to some studies [34,58]. However, others have extended this time until 12–14 days [59,60]. 

**Figure 10:**
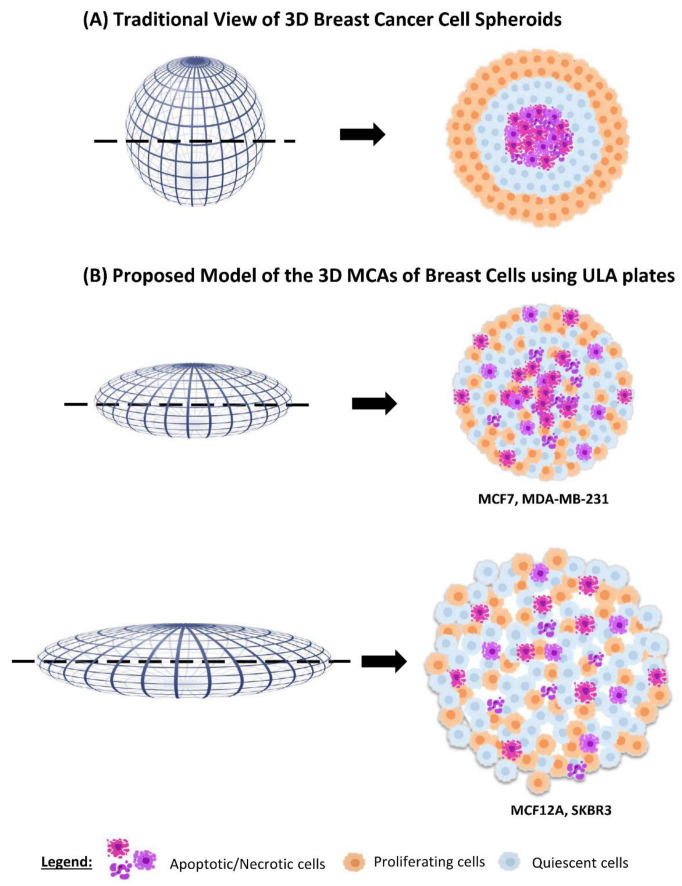
Schematic comparison of the traditional idealized three-layered distribution of dying, quiescent, and proliferating cells in spheroids, and the observed pattern in MCF7, MDA-MB-231, MCF12A, and SKBR3 MCAs obtained using ULA plates.

According to the authors that used similar methodologies, the 3D cultures were reported to be spherical or nearly spherical [14,61,62]. Contrarily, here the formed MCAs were ellipsoids or flattened discoid aggregates, as schematically represented in Figure 10. However, it is important to mention that the referred studies did not include histological analysis and only relied on observations with stereomicroscopy. At this level, our images are similar to previous findings [14,61,62]. Only when processing the MCAs for light and electron microscopy their real shape was observed, which varied according to the cell line, as well as their size and cellular compactness.

### 4.2. MCAs’ Compactness and Size

MCF7 and MDA-MB-231 formed compact MCAs, while SKBR3 and MCF12A formed loose MCAs. Previous studies with MCF7, MDA-MB-231 and SKBR3 also obtained 3D cultures with different compactness [22,49,63]. The more compact MCAs had a lower degree of flatness and smaller areas, while in loose MCAs the flatness was more pronounced and the areas were bigger (Figure 10). 

The observation of HE-stained sections validated the degree of compactness, showing cells tightly packed in compact MCAs. TEM also confirmed the status of intercellular adhesion, with desmosomes, tight junctions, and interdigitations being more developed and numerous in MCF7 MCAs, following prior studies [64,65]. Moreover, most MCF7 cells were positive for the E-cadherin cell adhesion protein, as in previous descriptions [66,67,68], agreeing well with the compactness of their 3D cultures [61]. The cell compaction in MDA-MB-231 was also explained by intercellular adhesions, but according to previous descriptions, it is not mediated by E-cadherin, otherwise by collagen I/integrin β1 interactions [61].

Most studies referred that MCF7 spontaneously formed compact spheroids [58,61,69,70], while one group described it as forming loose spheroids [15]. However, some authors report the addition of Matrigel or other viscosity raiser compound to the medium as a requirement for obtaining compact spheroids of MCF7 [22] and MDA-MB-231 [22,61,63]. This study produced similar results with the ULA plates without adding those compounds. Concerning the MDA-MB-231 cell line, our results are similar to some authors that obtained compact spheroids [69,71,72] but differ from others reporting loose aggregates [15,70]. These divergent results could be related to different methodologies to generate the 3D cultures, including different plate coatings, culture mediums with respective supplementations, and cell densities since all these factors can influence the formation of these models.

In the case of MCF12A, due to its shorter population doubling time (PDT) (19 h) compared to the other cell lines (MCF7, SKBR3, MDA-MB-231 (29, 30, and 38 h, respectively), half of the cell seeding density was used (40.000 cells/well) [73]; even so, the MCAs areas were bigger when compared to compact MCAs. This was probably due to the low compactness of its MCAs, confirmed in HE and TEM analysis, and not to its shorter PDT that, in theory, could contribute to a higher number of cells. Additionally, it is crucial to keep in mind that the available data about the PDT is relative to 2D cell cultures, and the behavior in 3D cell culture may not be the same, precisely due to the 3D nature of the cell-to-cell interactions.

### 4.3. Inner MCAs’ Structure

Many compact MCAs presented an apoptotic/necrotic core. The choice of this nomenclature “apoptotic/necrotic core” is associated with the fact that in HE and TEM there were cells with morphologies compatible with both apoptotic and necrotic cells. Here we confirmed apoptosis by ICC using an apoptotic marker (caspase-3 antibody), but according to the morphology observed in HE and electron microscopy, necrosis also occurs. 

Spheroids with diameters over 500 µm have been described as presenting a central necrotic area resultant from the depletion of oxygen, nutrients, and decreased pH [3,25]. Our MCAs were larger than 500 µm (except for MDA-MB-231), and only some compact MCAs presented apoptotic/necrotic cores.

Generally, our MCAs were larger than those reported before [15,57,62]. We opted to use high cell densities for obtaining large MCAs, which could better represent a model for microavascular tumors or micrometastasis [3,74]. Furthermore, the large size of the MCAs facilitated their visualization and manipulation for morphological analysis. 

Compared to the existing literature, the cell densities used in this study were, depending on the cell line, similar to or greater (up to about 60 times) than the ones used in other studies [15,58,62,70,71]. Nonetheless, the time for 3D formation and morphology was very similar. For example, Selby et al. (2017) plated only 5000 MCF7 cells/well using the same plates and equal time formation and obtained spheroids with diameters around 500 μm. Despite using 16 times more cells/well for the same cell line, the average diameters of the MCAs were only 1.4 times higher, around 700 μm. This leads us to hypothesize that cell density is not the most determinant factor for the MCAs size, contrarily it seems more relevant to the inner characteristics of the cell lines that lead to different degrees of aggregation.

Whatever the aggregate size, dying cells existed in the core. Smaller MCF7 spheroids have shown necrotic cores after 5 days in culture [22], and MDA-MB-231 also presented necrotic cores within 6 and 7 days in culture [75,76]; the diameters of these MCAs were in one case half the size of the ones in our study, showing that the size of the MCAs is not the only factor responsible for the apoptotic/necrotic core. Similarly, compact MCAs have been described as having a higher percentage of dead cells [61]. However, no differences in the % of caspase-3 positive cells were found in this study between the compact and loose MCAs. This was probably because the larger MCAs also presented low compactness allowing better diffusion of oxygen and nutrients.

Following the MCAs’ characterization, both light and electron microscopy disclosed some peculiar characteristics, especially in MCF7 MCAs, where it was detected the presence of acinar-like structures of different sizes that resemble the glandular acini of the mammary gland. The morphogenesis of this kind of acinar-like structure is said to involve the clearance of inner cells by apoptosis to allow lumen formation [67,77]. This is in line with our observations of cells with an apoptotic morphology and with the positive immunomarking for caspase-3 inside the lumen, equally reported by other authors [78]. Therefore, our results support the previous study of Amaral et al. [67], defending that the lumen formation was not due to the presence of substances that mimic the extracellular matrix (ECM). Nevertheless, the latter authors described that the luminal differentiation in spheroids only occurred after 50 days in culture. Herein, the acinar structures were detected on days 3 and 7 in culture, meaning that their differentiation, in the tested conditions, does not need so much time to occur. 

TEM observation revealed that the acinar-like structures presented cellular polarization with periluminal microvilli [65,66,79] and secretory vesicles [66], features that have been described earlier. Secretory vesicles (with β-casein, a protein of human milk) were attributed to the presence of egg white in unfertilized chicken eggs in the culture medium [66]. Differently, in our culture medium, there was no egg white. There is another major difference between our study and that of D’Anselmi et al. (2013), as they worked in monolayer cultures while we used 3D cultures, which are described as being more prone to promote cellular differentiation [16,80] and tissue recapitulation [81,82]. Still, regarding TEM observations, high amounts of glycogen existed in MCAs of MCF7, similar to previous descriptions [83]. The same applies to lipid droplets in MCAs of MCF7 and MDA-MB-231 [84]. Lastly, the presence of many dense bodies was characteristic in MCAs of MDA-MB-231, also following a previous observation in a 3D culture with the same cell line [85].

Regarding the formation of MCAs from the SKBR3 cell line, other researchers had already described the unsuccessful formation of 3D structures using ULA plates [69]. This might have happened because Piggott et al. [69] used a serum-free medium, and serum contains growth factors necessary to maintain cell proliferation; therefore, their withdrawal can cause cell arrest and apoptosis [86]. SKBR3 cells observed in scanning electron microscopy also revealed poor aggregation in 3D arrangements [87]. Nevertheless, SKBR3 could successfully form 3D cultures when a viscosity raiser was added to the medium [22,61]. The poor cell adhesion is correlated with the noted lack of expression of E-cadherin in SKBR3 MCAs, corroborating previous descriptions [49,68]. The low compactness can also be explained by the low expression of other adhesion molecules such as integrin β1 in this cell line [88].

MCF12A MCAs were the least compact aggregates in our study, which made them very difficult to manipulate without causing disaggregation. This study seems to be the first report describing the formation of MCF12A MCAs using ULA plates since the literature only reports MCF12A 3D cultures, using other techniques that include Matrigel [89,90]. In opposition to compact MCAs, the looser ones presented bigger areas, reflecting the intercellular spaces observed in HE staining and the decreased number of cellular adhesions in TEM analysis. 

In MCF12A, just a small number of cells stained positive for E-cadherin, forming clusters that appeared in the outer part of the MCAs. The literature relative to the expression of this protein is divergent since one study did not detect E-cadherin protein by Western blot [91], while another described this cell line as positive to E-cadherin using ICC [92]. However, the presented immunostaining was not in the membrane as expected for this marker and as we obtained in MCF12A (and MCF7) MCAs. 

In contrast to compact MCAs, there were no observed apoptotic/necrotic cores in loose MCAs. We hypothesize that the low compactness and the flattened shape increase the surface area and reduce the distance from the long axis to the MCAs center of mass, promoting better access to the nutrients and oxygen and, as a consequence, preventing the formation of a hypoxic central region [93]. 

The proportion of apoptotic and proliferating cells in the MCAs was evaluated using anti-caspase-3 and anti-Ki67 antibodies, respectively. In both compact and loose MCAs, there were proliferating cells throughout all the MCAs without preferential localization (see the schematic diagram in Figure 10). Even when an apoptotic/necrotic core was present, there were some Ki67 positive cells among the apoptotic cells, differing from the reported traditional diagrams of 3D cultures composed of three zones [2,13,16,25], in which proliferating cells were located solely in the outer part of the spheroids. The distribution of caspase-3 positive cells was overall similar to the one relative to Ki67, but there was a higher number of apoptotic cells in the apoptotic/necrotic core (whenever present). 

Compared to the typical schematic portrayal of 3D cultures, these changes in the distribution of caspase-3 and Ki67 positive cells may be attributed to the ellipsoid form of the MCAs. However, a similar random distribution of Ki67 positive cells was observed in MCF7 [22] (≈300–400 µm of diameter) and MDA-MB-231 spheroids (≈500–600 µm) [94], and other small spheroids from breast cell lines (≈300 µm) [95]. Thus, according to our results, the classical three-layered spheroid structure is too simplistic and does not represent the real structure of all the 3D cell cultures. Thus, at least for the four cell lines and used culture conditions studied here, we propose a new schematic model integrating the MCAs’ morphology, compactness, and distribution of proliferating and dead cells (Figure 10). 

The MCAs were also analyzed for the expression of epithelial (AE1/AE3) and mesenchymal (vimentin) antigens, both important when studying the epithelial–mesenchymal transition (EMT), where epithelial cells lose their polarity, change their shape, acquire motility, and start to express mesenchymal markers [96]. There has been a growing interest in developing drugs that target EMT [97,98], reinforcing the importance of studying epithelial and mesenchymal markers within the MCAs.

All MCAs stained positive for AE1/AE3. Our results are in accordance with the literature concerning MCF7 [99,100], SKBR3 [99,100], and MCF12A [92]. For MDA-MB-231, there is conflicting information in the published data, with some authors, like us, describing this cell line as being positive for CK19 [101], one of the various cytokeratins recognized by AE1/AE3 [102]. However, others reported that CK19 was not detected in this cell line by semi-quantitative RT-PCR and Western blot analyses [100] or that epithelial markers were weakly expressed in MDA-MB-231 [66].

Concerning vimentin, we have found strong positive immunomarking in MDA-MB-231 and MCF12A MCAs, and a few positive cells in MCF7. In general, our results corroborated the literature: MCF7 [66,99] and SKBR3 [99,103] had been described as negative for vimentin, while MDA-MB-231 [66,99] and MCF12A are reported as being positive for this marker [92,104]. However, we found a low number of vimentin-positive cells in MCF7 MCAs, suggesting that these cells have undergone EMT, a hypothesis supported by one study indicating that only around 5% of cells undergo EMT in 3D arrangement [105].

Another important characteristic of the breast cell lines is the expression of ER and PR hormonal receptors and the growth factor receptor HER-2, especially when studying the response or interaction of drugs with these receptors [106]. For MCF7, there is a broad consensus about its positivity to ER and PR [38,107,108], which is in perfect agreement with our results. Additionally, we unveiled for the first time that the positive cells are preferentially located in the outer part of the MCAs. For MCF12A, the literature presents contradictory data. Our results corroborate studies reporting that this cell line is negative for ER [39]. Other authors stated that it weakly expressed ER [109] or was non-responsive to estrogen [92]. Contrarily, some authors affirmed that MCF12A is ER/PR positive [40,110] and that it even highly expresses ERα and ERβ [89]. SKBR3 presented overexpression of HER-2 as it was supposed [38], with more than 80% of the cells in the MCAs showing a thick, circumferential uniform membrane staining [111].

## 5. Conclusions

The use of ULA plates was revealed to be a simple, fast, reproducible, and cost-effective technical option for generating and analyzing the MCAs of MCF7, MDA-MB-231, SKBR3, and MCF12A cell lines. The MCAs had an ellipsoid to discoid shape, either compact (MCF7 and MDA-MB-231) or loose and more flattened (MCF12A and SKBR3). Compact MCAs presented smaller areas with more cellular adhesions and apoptotic/necrotic cores. In looser MCAs, proliferating and apoptotic cells were more randomly distributed. MCF7 MCAs presented glandular breast differentiation features with the formation of acinar-like structures, with apical microvilli and adluminal accumulation of secretory vesicles. Given the conflicting data found in the literature, we recommend characterizing 3D models using different outputs. The presented cytological and ICC characterizations of MCAs from MCF7, SKBR3, MDA-MB-231, and MCF12A cell lines using ULA plates help to understand the strengths and weaknesses of this model but also give a baseline to the interpretation of experimental results, namely from cytotoxic assays and drug screening. The very inconsistent data in the literature concerning the characterization of 3D models of breast cell lines also reinforces the need to detail the used protocols and promote the use of standardized culture conditions, aiming for better replication.

## Figures and Tables

**Table 1:** Antibodies used in ICC characterization.

Antibody, Brand, City, Country	Host	Type, Clone	Dilution
Ki67, Biocare Medical, Pacheco, CA, USA	Rabbit	Monoclonal, SP6	1/200
Caspase-3 ab 13847, Abcam, Cambridge, UK	Rabbit	Polyclonal	1/5000
E- cadherin, Dako, Santa Clara, CA, USA	Mouse	Monoclonal, NCH-38	1/200
Cytokeratin, Cell Marque, Rocklin, CA, USA	Mouse	Monoclonal, AE1/AE3	1/1000
Vimentin, Novocastra, Milton Keynes, UK	Mouse	Monoclonal, V9	1/1600
Estrogen receptor (ER), Biocare Medical, Pacheco, CA, USA	Rabbit	Monoclonal, SP1	1/200
Progesterone receptor (PR), Biocare Medical, Pacheco, CA, USA	Rabbit	Monoclonal, 16	1/200
HER-2, Biocare Medical, Pacheco, CA, USA	Rabbit	Monoclonal, EP3	1/400

**Table 2:** Summary of the quantitative ICC analysis of the MCAs. Data (% of cells tagged) are given as mean ± standard deviation (3 independent replicates).

Markers	Cell Lines			
	MCF7	SKBR3	MDA-MB-231	MCF12A
Caspase-3	15 ± 5	26 ± 3	30 ± 11	21 ± 2
Ki67	38 ± 10 *	21± 5	26 ± 4	14 ± 10 *
AE1/AE3	76 ± 15	100	100	100
Vimentin	5 ± 2	0	100	100
E-cadherin	70 ± 4	0	0	11 ± 3
ER	53 ± 13	n.a.	n.a.	0
PR	49 ± 10	n.a.	n.a.	0
HER-2	n.a.	82 ± 1	n.a.	n.a.

* *p* < 0.05 (MCF7 vs. MCF12A); n.a: not applicable; no standard variation applies to 0 or 100%.

## Data Availability

The data presented in this study are available on request from the corresponding author on reasonable request.

## Supplementary Materials

The following supporting information can be downloaded at: https://www.mdpi.com/article/10.3390/toxics10080415/s1, Table S1: Tips and tricks for successful MCAs analysis from our experience. Figure S1. Representative images of the morphology (after 3 days in culture) of the 3D MCAs of MCF7, SKBR3, MDA-MB-231, and MCF12A cell lines seeded at three different cell densities.
