# Supplementary material for: Morphometrical, Morphological, and Immunocytochemical Characterization of a Tool for Cytotoxicity Research: 3D Cultures of Breast Cell Lines Grown in Ultra-Low Attachment Plates
Source: Toxics. 2022 Jul 24;10(8):415. doi: 10.3390/toxics10080415 (PMC9394479; doi:10.3390/toxics10080415)
Supplement: Supplementary file 1 [file toxics-10-00415-s001.zip › Figure S1.pdf]

toxics

MDPI

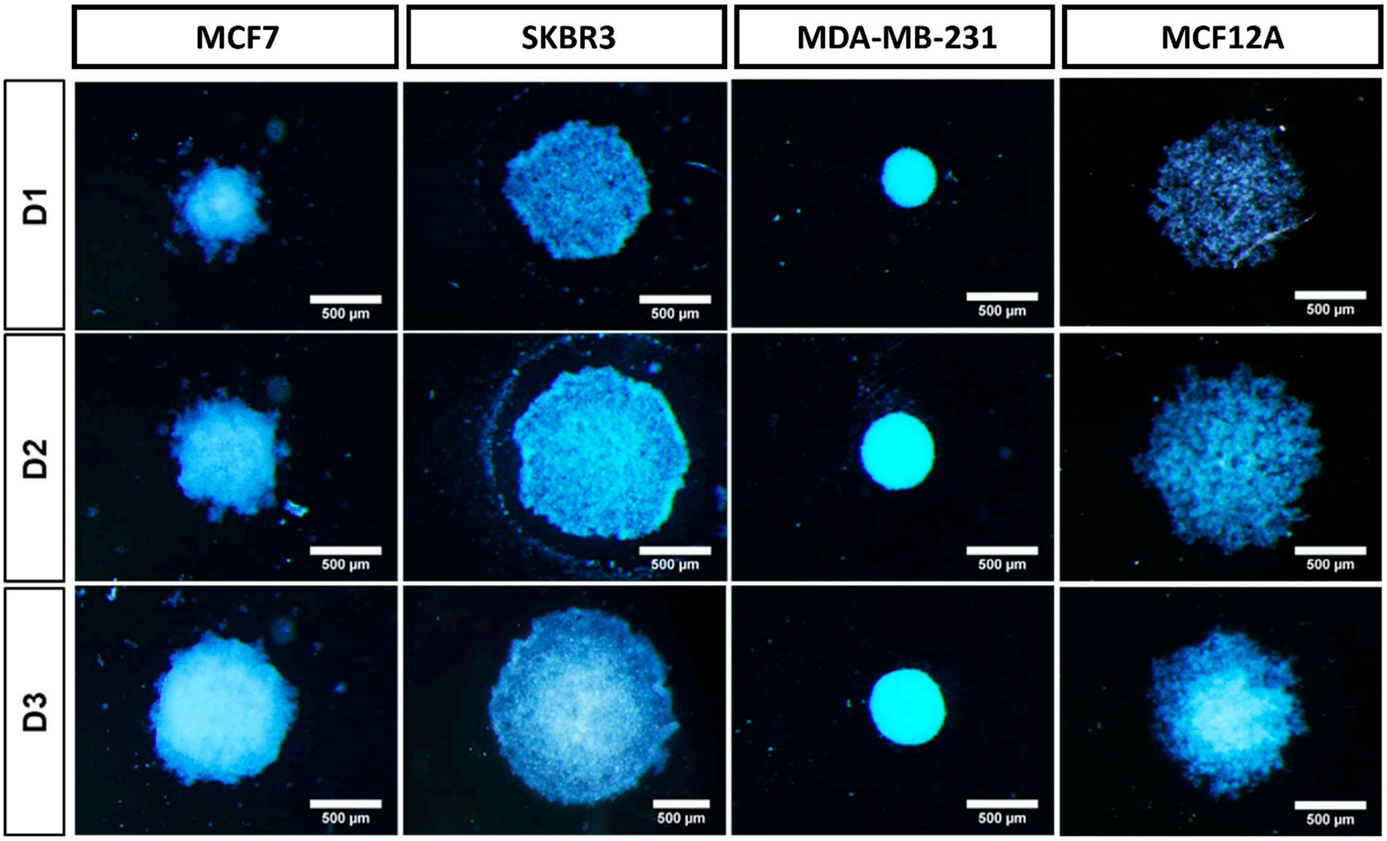

Figure S1. Representative images of the morphology (after 3 days in culture) of the 3D MCAs of MCF7, SKBR3, MDA-MB-231 and MCF12A cell lines seeded ate three different cell densities. MCF-7 and SKBR3 cells were seeded 10 x 10^4 cells/mL (D1), 20 x 10^4 cells/mL (D2) and 40 x 10^4cells/mL (D3). MDA-MB-231 cells were seeded at 20 x 10^4 cells/mL (D1), 40 x 10^4 cells/mL (D2) and 50 x 10^4 cells/mL (D3). MCF-12A cells were seeded at 5 x 10^4 cells/mL (D1), 10 x 10^4 cells/mL (D2), and 20 x 10^4 cells /mL (D3).
