# Supplementary material for: Morphometrical, Morphological, and Immunocytochemical Characterization of a Tool for Cytotoxicity Research: 3D Cultures of Breast Cell Lines Grown in Ultra-Low Attachment Plates
Source: Toxics. 2022 Jul 24;10(8):415. doi: 10.3390/toxics10080415 (PMC9394479; doi:10.3390/toxics10080415)
Supplement: Supplementary file 1 [file toxics-10-00415-s001.zip › Table S1.pdf]

toxics

MDPI

Supplementary Materials

Table S1: Tips and tricks of successful MCAs analysis by our experience.

**Problem**	**Recommendations**
Cells do not form MCAs	- Increase cell density.<br>- Centrifugation step is crucial to gather the cells.
MCAs are too small, easily lost during their manipulation	- Increase cell density.
Irregular MCAs formation due to the presence of fibres into the wells	- Use a laminar hood with good filters.<br>- Use synthetic lab coats.<br>- Keep the plate uncovered the least time possible.<br>- Plate the cells using multichannel pipettes to be faster.
MCAs lost or damaged during medium change	- Use a 100 µl micropipette and place the tip against the wall of the well to aspirate and replace the medium.<br>- Both aspiration and medium replacement should be done very slowly and carefully, as too much pressure can cause MCAs' damage.<br>- Lean the plate around 30º, since this helps seeing the MCAs and prevent their aspiration.
MCAs lost or damaged during harvesting for optical and electron microscopy	- Carefully remove the medium and replace it with the proper fixative, in the well. Incubate for 10 min before harvesting. Be careful not to leak the fixative

	into other wells destined for other types of proxies. In this step leave the lid open because fixatives release vapours than can interfere with other techniques.<br>- For harvesting use a 1000 µl micropipette with a sectioned tip that offers a larger circumference and prevents the MCAs' damage.<br>- The harvesting procedure should be performed slowly and carefully.<br>- Transfer to an Eppendorf tube containing the appropriate fixative.
MCAs lost or damaged during processing for optical and electron microscopy	- After the fixation step, embed the MCAs in histogel according to the supplier's instructions.
Difficult in finding MCAs during paraffin block sectioning	- Histogel containing the MCAs should be oriented during the embedding procedure. Sometimes it is necessary to remove the excess of histogel to facilitate the sectioning step.<br>- When sectioning, it is extremely important not to remove a large thickness of paraffin, as MCAs are not easy to see and therefore can be easily lost.<br>- Sections should be checked under the microscope to assess the presence of the MCA. Its observation is easier if the sections have been recently collected from the water.<br><br>Note: It is important to mention that to check the presence of an apoptotic core, it is necessary to section nearly all the MCA to assure that we have passed through the core
